# WISP1 induces ovarian cancer via the IGF1/αvβ3/Wnt axis

**DOI:** 10.1186/s13048-022-01016-x

**Published:** 2022-08-13

**Authors:** Yan Li, Fangfang Wang, Tianyi Liu, Nan Lv, Xiaolei Yuan, Peiling Li

**Affiliations:** 1 3th Ward of Obstetrics and Gynecology, The Second Affiliated Hospital of Harbin Medical University, Harbin, 150086 People’s Republic of China; 2 Prenatal Diagnosis Center, The Second Affiliated Hospital of Harbin Medical University, Harbin, 150086 People’s Republic of China; 3 1st Ward of Obstetrics and Gynecology, The Second Affiliated Hospital of Harbin Medical University, No. 246, Xuefu Road, Nangang District, Harbin, 150086 People’s Republic of China

**Keywords:** WISP1, Ovarian cancer, Epithelial-mesenchymal transition, IGF1, αvβ3, Wnt

## Abstract

**Background:**

This study intended to clarify the mechanisms by which WISP1-mediated IGF1/αvβ3/Wnt axis might affect the progression of ovarian cancer.

**Methods:**

Bioinformatics analysis was implemented for pinpointing expression of IGF1 and WISP1 which was verified through expression determination in clinical tissue samples and cells. Next, gain- or loss-of-function experimentations were implemented for testing CAOV4 and SKOV3 cell biological processes. The interaction between WISP1 and IGF1 was verified by co-immunoprecipitation and the molecular mechanism was analyzed. Finally, ovarian cancer nude mouse models were prepared to unveil the in vivo effects of WISP1/IGF1.

**Results:**

IGF1 and WISP1 expression was elevated in ovarian cancer tissues and cells, which shared correlation with poor prognosis of ovarian cancer sufferers. Elevated IGF1 induced malignant properties of ovarian cancer cells through activation of PI3K-Akt and Wnt signaling pathway. WISP1 was positively correlated with IGF1. WISP1 could enhance the interaction between IGF1 and αvβ3 to induce epithelial-mesenchymal transition. In vivo experiments also confirmed that upregulated WISP1/IGF1 induced tumorigenesis and metastasis of ovarian cancer cells.

**Conclusion:**

In conclusion, WISP1 can facilitate ovarian cancer by activating Wnt via the interaction between IGF1 and αvβ3.

**Supplementary Information:**

The online version contains supplementary material available at 10.1186/s13048-022-01016-x.

## Background

Ovarian cancer is the fifth leading cause of death related to cancer in women all over the world with the highest mortality in gynecological cancer [1]. It is known that ovarian cancer accounts for 2.5% of the incidence rate of female cancers [2]. Ovarian cancer is heterogeneous with the complicated and uncertain etiology, and risk factors for ovarian cancer include genetic risk, obesity, age, etc. [3]. Moreover, tumor-related deaths in patients with solid tumors have been reported to be attributed to metastasis or invasion instead of the primary tumor [4]. Epithelial-mesenchymal transition (EMT) is a well-characterized process involved in ovarian cancer cell metastasis [5]. Although the rapid advances have been done for ovarian cancer treatment, the clinical outcomes of patients with ovarian cancer are far from satisfactory [6]. Thus, it is urgent to determine the potential biomarker and molecular mechanism involved in ovarian cancer cells so as to offer new ways for ovarian cancer treatment.

WISP1, also known as CCN4 or Elm1, is a cysteine-rich, secreted matricellular protein that belongs to the CCN family [7]. Matricellular proteins are capable of modulating cellular responses, including cell growth and differentiation [8]. WISP1 exists in many parts of the body and is expressed in heart, kidney, lung, placenta, ovary, brain etc. [9]. A prior study has investigated that WISP1 is involved in multiple human cancers, such as lung cancer, breast cancer, and prostate cancer [10]. However, the role of WISP1 in ovarian cancer development is still unknown. In addition, The Cancer Genome Atlas (TCGA) database analysis revealed a putative relationship between WISP1 and insulin growth factor-1 (IGF1) in ovarian cancer. IGF1 is a circulating endocrine hormone, which is the main regulator of body growth and development [11]. The elevation of IGF1 contributes to the progression of cancer [12]. The available data support that IGF1 receptor (IGF1-R) is capable of controlling cell proliferation and metastasis in cancer [13]. Of note, IGF1 expression is increased in HTR8/SVneo cells treated with exo-Fatty acid-binding protein-4 and the proliferation and migration-related WISP1 is significantly expressed [14]. However, the mechanism by which WISP1 affects EMT in ovarian cancer involving the interplay with IGF1 is still poorly understood, which attracted our attention.

## Materials and methods

### Ethics statement

This study was started with the ratification of the Ethics Committee of The Second Affiliated Hospital of Harbin Medical University and carried out by referring to the *Declaration of Helsinki*. All subjects provided informed consent form. Animal experiments were ratified by the Ethics Committee of The Second Affiliated Hospital of Harbin Medical University.

### Bioinformatics analysis

UALCAN and GEPIA websites were employed to analyze the expression of IGF1 and WISP1 in adjacent normal tissues and ovarian cancer tissues, and the relationship between their expression with the prognosis and tumor stage of patients with ovarian cancer. IGF1-related genes and metastasis-related genes in ovarian cancer were analyzed by UALCAN website. The Venn tool was utilized to obtain the intersection genes to plot a Venn map. GO and KEGG enrichment analysis of the above intersection genes was carried out utilizing the ClueGO plug-in unit of Cytoscape software.

### Study subjects

Ovarian cancer tissues and adjacent normal tissues (at least 5 cm away from the tumor) were collected from 57 patients with ovarian cancer (aged 34–76 years old with a mean age of 55.07 ± 10.12 years) at The Second Affiliated Hospital of Harbin Medical University from September 2019 to October 2020. These patients had no other clinicopathological features and did not receive preoperative treatment, such as radiotherapy or chemotherapy. The histological diagnosis of ovarian cancer was evaluated in the light of the World Health Organization criteria. All collected tissues were immediately stored at − 80 °C for the following experiments.

### Cell culture and transfection

Human normal ovarian cell line IOSE80 (C1390) was purchased from Shanghai Zeye Biotech Co., Ltd. (Shanghai, China). Human ovarian cancer cell lines CAOV4 (HTB-76) and SKOV3 (HTB-77) were procured from COBIOER Company (Nanjing, China). Human ovarian cancer cell line CoC1 was bought from Procell Life Science & Technology Co., Ltd. (Wuhan, Hubei, China). These cells were incubated in 1640 medium replenishing 10% fetal bovine serum (FBS, Gibco Company, Grand Island, NY), 10 μg/mL streptomycin, and 100 U/mL penicillin (Gibco) and placed in an incubator (Thermo Fisher Scientific Inc., Waltham, MA) at 37 °C with 5% CO_2_.

Some cells were treated with IGF1 (GF306, Sigma-Aldrich, St. Louis, MO) at concentrations of 0.1 nmoL, 1 nmoL and 10 nmoL, respectively. Logarithmically growing cells were trypsinized and seeded in a 6-well plate (1 × 10^5^ cells/well) and cultured for 24 h. Upon75% confluence, the cells were treated with dimethyl sulfoxide, IGF1, negative control (NC) for short hairpin RNA (shRNA) (shNC), shRNA against IGF1 (shIGF1), and shWISP1 with the help of Lipofectamine 2000 (Invitrogen, Carlsbad, California). The plasmids of shNC, shIGF1, and shWISP1 were purchased from Sigma. The sequences of shRNA are shown in Supplementary Table 1.

### Reverse transcription quantitative polymerase chain reaction (RT-qPCR)

Total RNA was extracted utilizing TRIzol reagent (15,596,026, Invitrogen) and reversely transcribed into complementary DNA (cDNA) with the help of a PrimeScript RT reagent Kit (RR047A, Takara, Japan). RT-qPCR was conducted by means of Fast SYBR Green PCR kit (Applied Biosystems, Carlsbad, CA) and ABI PRISM 7300 RT-PCR system (Applied Biosystems). Three replicates were prepared for each well. GAPDH was adopted as the normalizer for mRNA. The 2^-ΔΔCt^ method was employed to quantify relative expression of target genes. The primers are summarized in Supplementary Table 2.

### Cell proliferation analysis

Cell Counting Kit-8 (CCK-8) (Shanghai Beyotime Biotechnology Co., Ltd., Shanghai, China) was employed for testing cell viability. The absorbance at the wavelength of 452 nm was tested utilizing Microplate reader.

### Scratch test

Cells were added into the 6-well plate (5 × 10^5^ cells/well) and cultured overnight in serum-free medium. The horizontal lines were constructed evenly every 0.5–1 cm on the bottom surface of the 6-well plate using a sterile 10 μL pipette with the help of ruler and marker, at least five lines across each well. The images were gained under microscope and the cell migration was observed.

### Transwell assay

Transwell invasion assay was implemented with a Transwell system (Corning, USA) pre-coated with Matrigel (BD Biosciences, San Jose, CA) [15]. The stained invasive cells were counted under an inverted light microscope (Carl Zeiss, Jena, Germany) in at least 5 randomly-selected fields.

### Western blot analysis

Cells were lysed with enhanced radio immunoprecipitation assay lysis appended to protease inhibitor (BOSTER Biological Technology Co., Ltd., Wuhan, Hubei, China). The protein concentration was measured by the Bicinchoninic Acid Assay Kit (BOSTER). Following electrophoresis separation, the protein was transferred onto polyvinylidene difuoride membrane. After blocked with 5% bovine serum albumin for 2 h to inhibit non-specific binding, membranes were incubated with diluted primary antibodies (Supplementary Table 3) overnight at 4 °C. The membranes were then incubated with the horseradish peroxidase (HRP)-labeled secondary antibody (1: 2000) (Supplementary Table 3) at room temperature for 1 h. The membrane was developed with ECL working solution (EMD Millipore) for 1 min with the results analyzed with ImageJ software.

### Co-immunoprecipitation (co-IP)

For co-IP, a certain amount of cell lysate was incubated with 30 μL Protein A&G Agarose and 1 μg rabbit IgG or IGF1 primary antibody at 4 °C overnight. After incubation, the supernatant was removed through centrifugation. The 0.5 M Nacl lysis buffer was used to rinse the lower layer twice, and then the sample was boiled at 97 °C for 7 min, followed by Western blot analysis. Antibodies used in the experiment are shown in Supplementary Table 3.

### Xenograft model

Healthy female nude mice (aged 4–5 weeks) (Vital River Laboratories, Beijing, China) were raised in separate SPF animal laboratory with humidity of 60–65% at 22–25 °C. The mice were acclimated for one week before experiment.

The CAOV4 cell suspension (1 × 10^5^ cells/100 μL) harboring NC for gene overexpression (OE-NC) or overexpression of WISP1 (OE-WISP1) was injected subcutaneously into the nude mice during the feeding process, followed by intraperitoneal injection of IGF1 antibody or phosphate buffered saline (PBS), once every three days. The tumor volume was measured by Vernier calipers on the 7th day, and then once every 7 days. The nude mice were euthanized after 5 weeks to calculate the tumor weight.

In order to monitor the lung metastasis, CAOV4 cell suspension (1 × 10^6^ cells/100 μL) harboring OE-NC or OE-WISP1 was injected into the nude mice via tail vein during the feeding process, followed by intraperitoneal injection of IGF1 antibody or PBS, once every three days. After 4 weeks, lung metastasis was observed.

### Hematoxylin and eosin (HE) staining

The HE staining kit (G1120, Beijing Solarbio Science & Technology Co., Ltd., Beijing, China) was employed for this assay [16]. The changes of tumor morphology were observed under a light microscope.

### Immunohistochemistry (IHC) assay

The sections to be tested were heated at 60 °C for 20 min, immersed in xylene for 15 min and rehydrated with 100, 95, 90, 85 and 80% ethanol. Each section was incubated with 3% H_2_O_2_ for 15 min at room temperature to remove endogenous peroxidase. The tissues were treated with 0.01 mol/L citrate buffer (pH 6.0) in microwave oven for 10 min for antigen retrieval. After cooling, the sections were sealed with 5% normal goat serum for 15 min at ambient temperature and incubated with antibodies against E-cadherin, ZO-1, N-cadherin and SNAIL at 4 °C overnight, followed by addition of biotinylated goat anti-rabbit IgG and incubation with HRP-streptomycin for 15 min. Subsequently, the sections were treated with diaminobenzidine solution for 3–5 min, counterstained with hematoxylin for 1–3 min, dehydrated, and sealed with neutral balm. The above steps were repeated with 0.1 mol/L PBS (pH 7.4) as NC. Supplementary Table 3 displays the detailed information for the used antibodies.

### Statistical analysis

Data analysis was implemented utilizing the SPSS 21.0 software (IBM, Armonk, NY). All measurement data are concluded as mean ± standard deviation. Paired *t*-test was employed for comparisons between adjacent normal tissues and ovarian cancer tissues. The comparison between two groups was analyzed by independent sample *t*-test. For multiple independent groups, one-way analysis of variance (ANOVA) with post hoc Tukey’s test was used. Two-way ANOVA was utilized for the comparison of data at different time points (cell viability), and repeated measures ANOVA was employed for tumor volume analysis in combination with post hoc Bonferroni test. Pearson’s correlation analysis was implemented for testing the relationship between indexes. *p* < 0.05 considered significant.

## Results

### IGF1 is upregulated in ovarian cancer tissues in association with poor prognosis of ovarian cancer patients

**Fig. 1:**
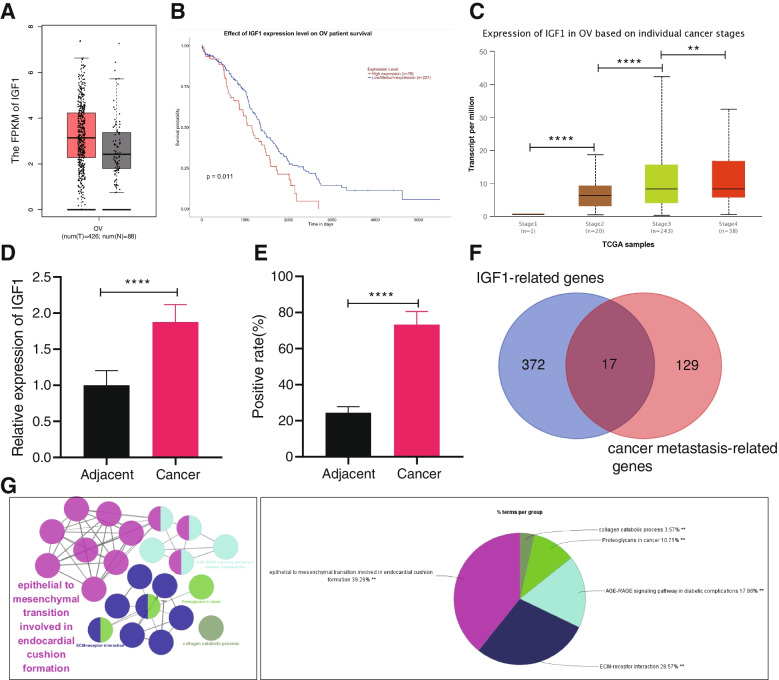
IGF1 expression is elevated in ovarian cancer, which is related to the dismal prognosis of ovarian cancer patients. **A** IGF1 expression in adjacent normal tissues and ovarian cancer tissues analyzed using GEPIA website (* *p* < 0.05 vs. adjacent normal tissues). The abscissa OV represents ovarian cancer, T represents tumor tissue samples (red box), and N represents adjacent normal tissue samples (gray box). **B** Relationship between IGF1 expression and prognosis of ovarian cancer patients analyzed using UALCAN website. **C** The relationship between IGF1 expression and tumor grade of patients with ovarian cancer analyzed using UALCAN website (** *p* < 0.01, **** *p* < 0.0001). **D** IGF1 expression in ovarian cancer tissues and adjacent normal tissues determined by RT-qPCR (*n* = 57; **** *p* < 0.0001, vs. adjacent normal tissues). **E** IGF1 expression in ovarian cancer tissues and adjacent normal tissues determined by IHC (*n* = 57; **** *p* < 0.0001, vs. adjacent normal tissues). **F** Venn diagram of IGF1 related genes and tumor metastasis-related genes analyzed by UALCAN website, followed by intersection. Blue circles represent 389 genes associated with IGF1 expression, and red circles represent 146 tumor metastasis-related genes. **G** GO and KEGG enrichment analysis of key genes performed by ClueGO (According to KappaScore, they were divided into five groups and expressed in five different colors). Data comparisons between adjacent normal tissues and ovarian cancer tissues were analyzed by paired *t*-test

IGF1 induces EMT in diverse diseases, such as breast cancer, prostate cancer, and gastric cancer, thereby enhancing the invasion and metastasis of cells [17]. Here, this study aimed to explore whether IGF1 also affects ovarian cancer. Through GEPIA and UALCAN databases, we found that IGF1 was elevated in ovarian cancer tissues (Fig. 1A), and the higher the IGF1 expression corresponded to lower survival rate of patients with ovarian cancer (Fig. 1B). In addition, the results of UALCAN database showed that the expression of IGF1 was increased over the clinical staging of ovarian cancer (Fig. 1C).

For determining the clinical significance of *IGF1*, the 57 cases of ovarian cancer tissues and adjacent normal tissues were analyzed (Supplementary Table 4). RT-qPCR and IHC showed increased IGF1 expression in ovarian cancer tissues (Fig. 1D, E). The genes positively related to IGF1 expression in ovarian cancer were analyzed by UALCAN database, revealing 389 genes, which were further intersected with 146 tumor metastasis-related genes recommended by UALCAN database. There were 17 candidate genes obtained in the intersection (Fig. 1F). The 17 candidate genes were summarized (Supplementary Table 5). GO and KEGG enrichment analysis using ClueGO plug-in displayed that 17 candidate genes were highly associated with EMT signaling pathway (Fig. 1G). Therefore, we inferred that the expression of IGF1 in ovarian cancer was significantly related to EMT signaling pathway. Taken together, the above results suggested that IGF1 was abundantly expressed in ovarian cancer and was associated with poor prognosis of ovarian cancer patients.

### IGF1 promotes migration, invasion and EMT of ovarian cancer cells

**Fig. 2:**
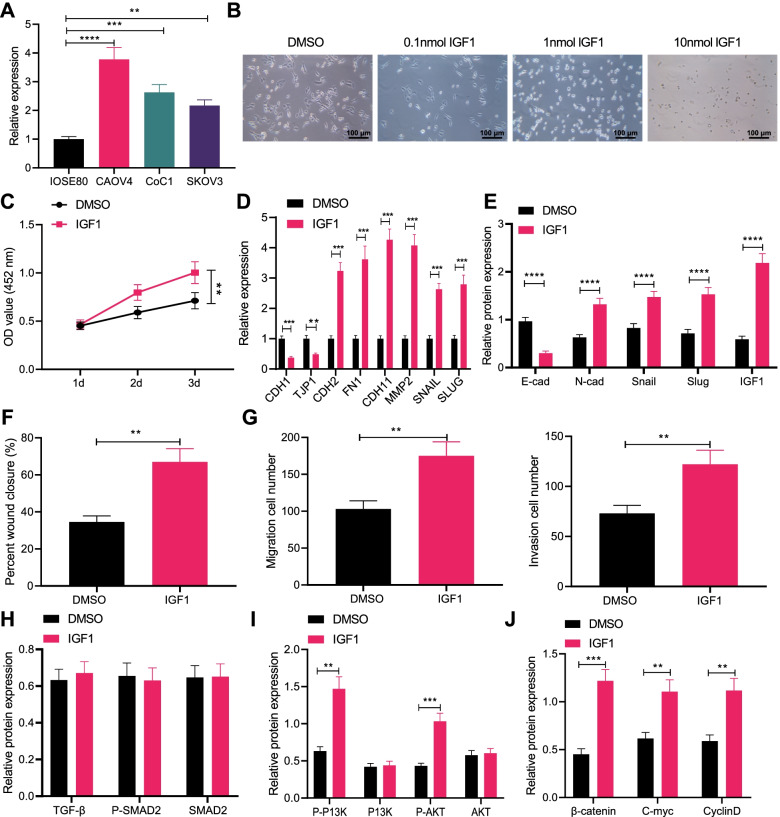
IGF1 induces migration, invasion and EMT of ovarian cancer cells. **A** IGF1 expression in IOSE80, CAOV4, CoC1 and SKOV3 cells determined by RT-qPCR. **B** Effects of different concentrations of IGF1 on the cell morphology, scale bar: 100 μm. SKOV-3 cells were treated with 1 nmol IGF1. **C** SKOV-3 cell viability detected by CCK-8 assay. **D** mRNA levels of EMT-related factors CDH1, TJP1, CDH2, FN1, CDH11, MMP2, SNAIL and SLUG in SKOV-3 cells determined by RT-qPCR. **E** Protein levels of EMT-related factors E-cadherin, N-cadherin, Snail and Slug in SKOV-3 cells determined by Western blot analysis. **F** SKOV-3 cell migration detected by scratch test. **G** SKOV-3 cell invasion detected by Transwell assay. **H** Expression of the TGF-β signaling pathway-related proteins TGF-β, P-SMAD2 and SMAD2 in SKOV-3 cells detected by Western blot analysis. **I** Expression of the PI3K-AKT signaling pathway-related proteins P-PI3K, PI3K, P-AKT and AKT in SKOV-3 cells detected by Western blot analysis. **J** Expression of the Wnt signaling pathway-related proteins β-catenin, C-myc and CyclinD in SKOV-3 cells detected by Western blot analysis. Data are shown as the mean ± standard deviation of three technical replicates. Data comparisons between two groups were analyzed by independent sample *t*-test. Data comparisons among multiple groups were analyzed by one-way ANOVA with Tukey’s post hoc test. Comparisons of data at different time points were analyzed by two-way ANOVA with Bonferroni post hoc test. ** *p* < 0.01; *** *p* < 0.001; **** *p* < 0.0001

RT-qPCR for quantification of IGF1 expression in human normal ovarian cell line IOSE80 and ovarian cancer cell lines (CAOV4, CoC1 and SKOV3) presented that IGF1 expression was elevated in CAOV4, CoC1 and SKOV3 cells when compared with that in IOSE80 cell line, among which the highest IGF1 expression was detected in CAOV4 cell line and the lowest IGF1 expression was detected in SKOV3 cell line (Fig. 2A). The effect of IGF1 on biological functions of ovarian cancer cells was investigated in SKOV3 cells. SKOV3 cells were treated with different concentrations of IGF1 for 72 h to observe the morphological changes. After 1 nmol and 10 nmol IGF1 treatment, SKOV-3 cells obtained the characteristics of mesenchymal cells (Fig. 2B). Therefore, *IGF1* at a concentration of 1 nmol was selected for subsequent experiments.

CCK8 assay exhibited that cell proliferation was promoted in SKOV-3 cells treated with IGF1 (Fig. 2C). RT-PCR showed that the expression of epithelial markers CDH1 and TJP1 decreased, while the expression of mesenchymal markers CDH2, FN1, CDH11, MMP2, SNAIL and SLUG increased in SKOV-3 cells treated with IGF1 (Fig. 2D), which was verified by Western blot analysis (Fig. 2E, Supplementary Fig. 1A). Scratch test and Transwell assay exhibited that treatment with IGF1 promoted the migratory and invasive capabilities of SKOV-3 cells (Fig. 2F, G).

As evidence indicated, TGF-β, PI3K-AKT, and Wnt signaling pathways can mediate EMT [18–20], while the regulatory mechanism of IGF1 in ovarian cancer is still unclear. Therefore, to explore whether IGF1 affects the TGF-β, PI3K-AKT and Wnt signaling pathways, Western blot analysis was conducted, which exhibited that IGF1 exerted no effects on the TGF-β signaling pathway, but it activated the PI3K-AKT and Wnt signaling pathways (Fig. 2H-J, Supplementary Fig. 1B-D). Conclusively, IGF1 could enhance the migration, invasion and EMT of ovarian cancer cells, and activated the PI3K-AKT and Wnt signaling pathways.

### Silencing of IGF1 inhibits migration, invasion and EMT of ovarian cancer cells

**Fig. 3:**
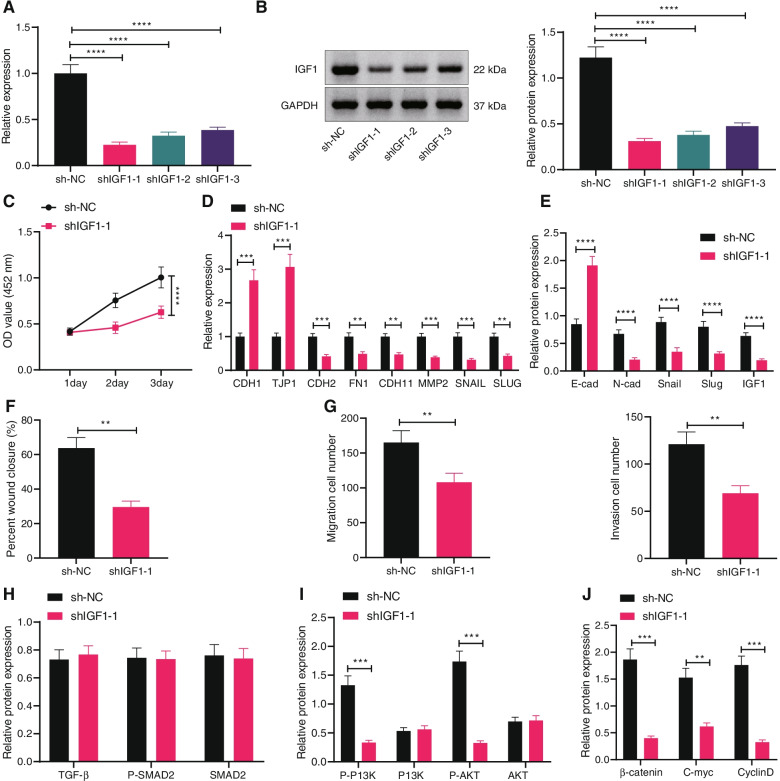
IGF1 knockdown represses migration, invasion and EMT of ovarian cancer cells. **A** The silencing efficiency of shRNAs targeting IGF1 in CAOV4 cells detected by RT-qPCR. **B** The silencing efficiency of shRNAs targeting IGF1 in CAOV4 cells detected by Western blot analysis. CAOV4 cells were transduced with sh-IGF1. **C** CAOV4 cell viability detected by CCK-8 assay. **D** mRNA levels of EMT-related factors CDH1, TJP1, CDH2, FN1, CDH11, MMP2, SNAIL and SLUG in CAOV4 cells determined by RT-qPCR. **E** Protein levels of EMT-related factors E-cadherin, N-cadherin, Snail and Slug in CAOV4 cells determined by Western blot analysis. **F** CAOV4 cell migration detected by scratch test. **G** CAOV4 cell invasion detected by Transwell assay. **H** Expression of the TGF-β signaling pathway-related proteins TGF-β, P-SMAD2 and SMAD2 in CAOV4 cells detected by Western blot analysis. **I** Expression of the PI3K-AKT signaling pathway-related proteins P-PI3K, PI3K, P-AKT and AKT in CAOV4 cells detected by Western blot analysis. **J** Expression of the Wnt signaling pathway-related proteins β-catenin, C-myc and CyclinD in CAOV4 cells detected by Western blot analysis. Data are shown as the mean ± standard deviation of three technical replicates. Data comparisons between two groups were analyzed by independent sample *t*-test. Data comparisons among multiple groups were analyzed by one-way ANOVA with Tukey’s post hoc test. Comparisons of data at different time points were analyzed by two-way ANOVA with Bonferroni post hoc test. ** *p* < 0.01; *** *p* < 0.001; **** *p* < 0.0001

We then pinpointed the effect of endogenous IGF1 on ovarian cancer cell biological functions. Three shRNAs were packaged by lentivirus to transduce CAOV4 cells to construct an IGF1 silencing cell line. As validated by RT-qPCR and Western blot analysis, the efficiency of shIGF1–1 was the highest, and the cell line stably transfected with shIGF1–1 was used for subsequent experimentations (Fig. 3A, B).

CCK-8 assay (Fig. 3C), scratch test (Fig. 3F), and Transwell assay (Fig. 3G) displayed that depleted IGF1 suppressed CAOV4 cell proliferative, migratory, and invasive capacities. In addition, silencing of IGF1 increased the expression of epithelial markers and downregulated that of mesenchymal markers in CAOV4 cells (Fig. 3D, E, Supplementary Fig. 1E), illustrating that silencing of IGF1 exerted inhibitory effect on EMT of ovarian cancer cells. Further, silencing IGF1 did not affect the TGF-β signaling pathway, but inhibited the PI3K-AKT and Wnt signaling pathways (Fig. 3H-J, Supplementary Fig. 1F-H). The aforementioned results demonstrated that silencing of IGF1 could arrest the migration, invasion and EMT of ovarian cancer cells, and inactivated the PI3K-AKT and Wnt signaling pathways.

### WISP1 promotes the interaction between IGF1 and αvβ3

**Fig. 4:**
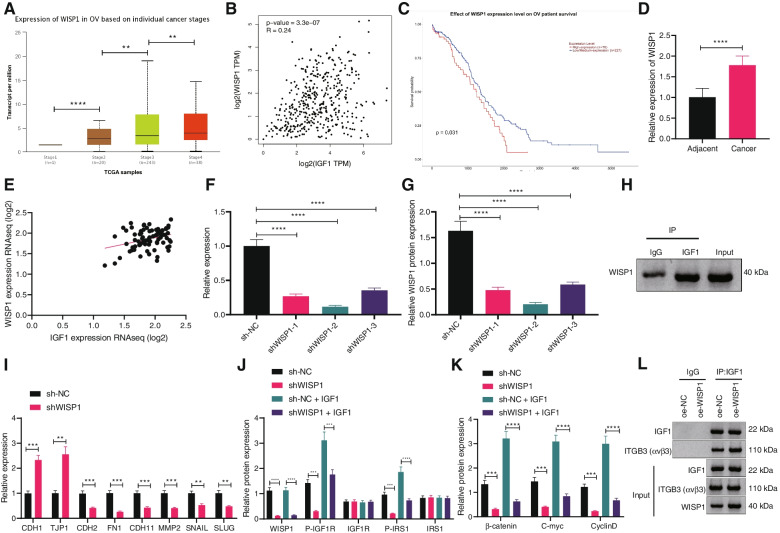
Effect of WISP1 on downstream signal transduction of IGF1. **A** Relationship between WISP1 expression and tumor stage of ovarian cancer analyzed by UALCAN website. **B** Expression of WSIP1 and IGF1 in ovarian cancer tissues analyzed by TCGA database. **C** Relationship between WISP1 expression and prognosis of patients with ovarian cancer analyzed by UALCAN website. **D** WISP1 expression in adjacent normal tissues and ovarian cancer tissues determined by RT-qPCR (*n* = 57; **** *p* < 0.0001, vs. adjacent normal tissues). **E** The expression of WISP1 and IGF1 in ovarian cancer tissues (*n* = 57) was analyzed by Pearson’s correlation analysis. **F** Silencing efficiency of shRNA targeting IGF1 detected by RT-qPCR. **G** Silencing efficiency of shRNAs targeting IGF1 detected by Western blot analysis. **H** The interaction between IGF1 and WISP1 detected by Co-IP. Cells were transduced with sh-WISP1 and/or IGF1. **I** Expression of EMT-related factors CDH1, TJP1, CDH2, FN1, CDH11, MMP2, SNAIL and SLUG in cells determined by RT-qPCR (*p < 0.05 vs. cells transduced with sh-NC). **J** Protein levels and phosphorylated levels of IGF1R and IRS1 in cells determined by Western blot analysis. **K** Protein levels of the Wnt signaling pathway-related proteins β-catenin, C-myc and CyclinD in cells determined by Western blot analysis. **L** The interaction between αvβ3 and IGF1 detected by Co-IP. Data are shown as the mean ± standard deviation of three technical replicates. Data comparisons between adjacent normal tissues and ovarian cancer tissues were analyzed by paired *t*-test. Data comparisons between two groups were analyzed by independent sample *t*-test. Data comparisons among multiple groups were analyzed by one-way ANOVA with Tukey’s post hoc test. Pearson’s correlation analysis was carried out to determine the relationship between indexes. *** *p* < 0.001; **** *p* < 0.0001

To discuss the molecular mechanism of IGF1 promoting ovarian cancer cell migration, the genes related to *IGF1* expression and EMT pathway in ovarian cancer were further analyzed from UALCAN database. The results clarified that the expression of Wnt signaling pathway-related protein WISP1 was correlated with tumor stage and WISP1 expression was elevated over the clinical stages of tumors (Fig. 4A). TCGA database analysis displayed that WISP1 expression was positively correlated with IGF1 protein level (Fig. 4B). UALCAN database showed that the prognosis of ovarian cancer patients with high WISP1 expression was poor (Fig. 4C). RT-qPCR revealed elevated WISP1 in ovarian cancer tissues (Fig. 4D). Pearson’s correlation analysis exhibited that WISP1 expression was positively correlated with IGF1 expression in ovarian cancer tissues (Fig. 4E).

Three shRNA sequences targeting WISP1 were constructed and transduced into CAOV4 cells to unravel the action of WISP1 and IGF1 in ovarian cancer. RT-qPCR and Western blot analysis exhibited that the efficiency of shWISP1–2 was the highest (Fig. 4F, G, Supplementary Fig. 1I) and used for subsequent experiments. Co-IP assay suggested that IGF1 was interacted with WISP1 (Fig. 4H). Moreover, silencing of WISP1 resulted in increased expression of epithelial markers and decreased that of mesenchymal markers (Fig. 4I).

Signal transduction of IGF1 in cells was achieved by activating the downstream IGF1R/IRS1 axis. In order to further study whether WISP1 mediated the activation of IGF1 signal axis, Western blot analysis showed that depleted WISP1 effectively inhibited the protein levels and phosphorylation of IGF1R and IRS1 and suppressed IGF1-activated Wnt signaling pathway. Moreover, the inhibitory action was found to be restored by IGF1 (Fig. 4J, K, Supplementary Fig. 1 J, K). Several studies have deciphered that αvβ3 promotes IGF1/IGF1R axis by interacting with IGF1, and WISP1 could regulate αvβ3 [21, 22]. Therefore, we further explored whether WISP1 affected the interaction between IGF1 and αvβ3, and co-IP displayed that overexpressed WISP1 enhanced the interaction between IGF1 and αvβ3 (Fig. 4L). Given the aforementioned experimental data, it can be concluded that WISP1 promoted the interaction between IGF1 and αvβ3.

### WISP1 promotes the tumorigensis and metastasis of ovarian cancer in vivo via IGF1

**Fig. 5:**
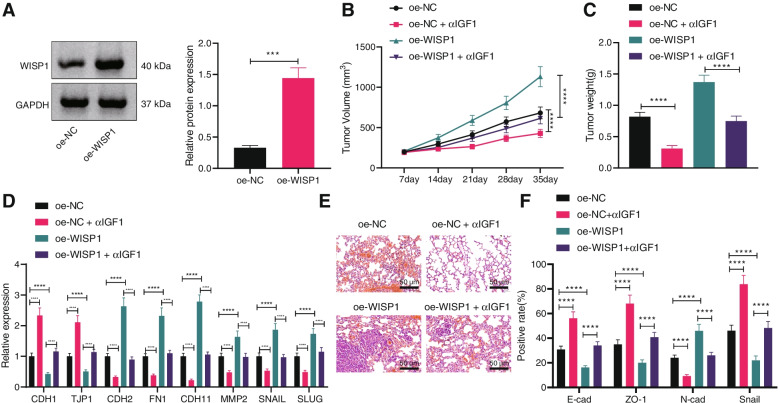
WISP1 favors the tumorigensis and metastasis of ovarian cancer in vivo via IGF1*.* Nude mice were injected with CAOV4 cells transduced with OE-WISP1 and treated with IGF1 antibody (*n* = 10). A, WISP1 protein level in CAOV4 cells determined by Western blot analysis. B, Tumor volume in nude mice. C, Tumor weight in nude mice. D, Expression of EMT-related factors CDH1, TJP1, CDH2, FN1, CDH11, MMP2, SNAIL and SLUG in lung tissues of nude mice determined by RT-qPCR. E, Lung metastases in nude mice detected by HE staining, scale bar: 50 μm. F, Protein levels of EMT-related factors E-cadherin, ZO-1, N-cadherin and SNAIL in lung tissues of nude mice determined by IHC. Data are shown as the mean ± standard deviation. Data comparisons between two groups were analyzed by independent sample *t*-test. Data comparisons among multiple groups were analyzed by one-way ANOVA with Tukey’s post hoc test. Comparisons of data at different time points were analyzed by two-way ANOVA with Bonferroni post hoc test. ** *p* < 0.01; *** *p* < 0.001; **** *p* < 0.0001

To further investigate the function of *WISP1/IGF1* in vivo, xenograft tumor mouse models were constructed. CAOV4 cells with stable expression of *WISP1* were constructed with the overexpression efficiency confirmed by Western blot analysis (Fig. 5A). Next, CAOV4 cells were injected into the armpit of nude mice. The morphology and size of transplanted tumor were observed, which presented that the volume and weight of transplanted tumor increased in nude mice injected with CAOV4 cells harboring OE-WISP1, while contrary results were seen upon IGF1 antibody (Fig. 5B, C). RT-qPCR exhibited that restored *WISP1* inhibited the expression of E-cadherin and ZO-1 and promoted the expression of N-cadherin and SNAIL, while anti-IGF1 treatment brought about opposite findings (Fig. 5D).

CAOV4 cells were then injected into nude mice via tail vein to investigate the effect of the WISP1/IGF1 axis on the in vivo metastasis of ovarian cancer cells. HE staining presented obvious metastases in the ovarian tissues of nude mice in presence of overexpression of WISP1 while opposite results were observed in presence of anti-IGF1 (Fig. 5E). HC displayed that after anti-IGF1 treatment, the expression of E-cadherin and ZO-1 increased, while that of N-cadherin and SNAIL decreased yet overexpressed *WISP1* induced opposite changing tendency (Fig. 5F). It can be concluded that WISP1 facilitated the tumorigensis and metastasis of ovarian cancer in vivo via IGF1.

## Discussion

Ovarian cancer is one of the gynecological malignancies, which leads to thousands of deaths in women around the world [23]. In spite of great progress in early detection and systematic treatment in the past few years, the 5-year survival rate of patients with ovarian cancer is still very low [2]. Here, we focused on the effect of WISP1, IGF1, αvβ3, and Wnt on ovarian cancer and the underlying mechanisms. Data obtained in our study demonstrated that WISP1 exerted facilitated properties on ovarian cancer via enhancement of interaction between IGF1 and αvβ3.

Our initial observations revealed elevated WISP1 in ovarian cancer tissues, which was related to the poor prognosis of patients with ovarian cancer. Aberrant expression of WISP1 is implicated in multiple pathologies, including cancer [7]. Available evidence has proved that *WISP1* is expressed in the ovaries [9]. Both in vitro and in vivo experiments revealed that upregulated *WISP1* enhanced the ovarian cancer cell proliferation, migration, invasion, and EMT to facilitate the progression of ovarian cancer. EMT has been demonstrated to be correlated with the invasion and metastasis of ovarian cancer, highly suggestive of poor prognosis [24]. As recently studied, the acquisition of invasive properties by ovarian cancer cells is accompanied by a reduction of epithelial features and an elevation of mesenchymal features [5]. WISP1 is also reported to be involved in epithelial-mesenchymal crosstalk [25]. Wu et al. have explored that WISP1 expression is elevated in colon cancer tissues, and WISP1 enhances the progression of colon cancer by enhancement of cell proliferation [10]. However, there are few studies on the role of WISP1 in ovarian cancer development. In this study, we confirmed that overexpressed WISP1 accelerated EMT of ovarian cancer cells.

Moreover, the obtained findings here proved that WISP1 was positively correlated with IGF1, which was also upregulated in ovarian cancer tissues, and IGF1 promoted malignant characteristics of ovarian cancer cells. IGF1 mediates apoptosis, migration, and differentiation of mammary epithelial cells [26]. A prior study has exhibited that elevated IGF1-R enhances proliferation and metastasis of cancer cells [13]. Additionally, WISP1 facilitated the interaction between IGF1 and αvβ3. Integrin αvβ3 plays a role in the IGF1 signaling, suggesting that IGF1 directly binds to αvβ3 [21]. It has also been verified that WISP1 regulates αvβ3 integrin signaling [22]. These findings support that WISP1 facilitated the interaction between IGF1 and αvβ3 to induce ovarian cancer.

Furthermore, the present study also indicated that silencing of IGF1 suppressed PI3K-AKT and Wnt signaling pathways. The importance of PI3K-Akt signaling pathway in ovarian cancer has been well-characterized [27, 28]. Wnt signaling is considered as a complex and fundamental developmental pathway that is dysregulated in various human malignancies, including ovarian cancer [29]. Wnt exerts great effects on the development of ovarian cancer by facilitating EMT, metastasis, and tumor angiogenesis of cancer stem cells [30]. Activated Wnt may result in EMT, thereby promoting the malignant properties of ovarian cancer cells [31]. As previously reported, IGF1 acts as an inducer for human ovarian cancer by activating the PI3K/AKT/mTOR signaling [32], which is in line with our finding.

## Conclusions

**Fig. 6:**
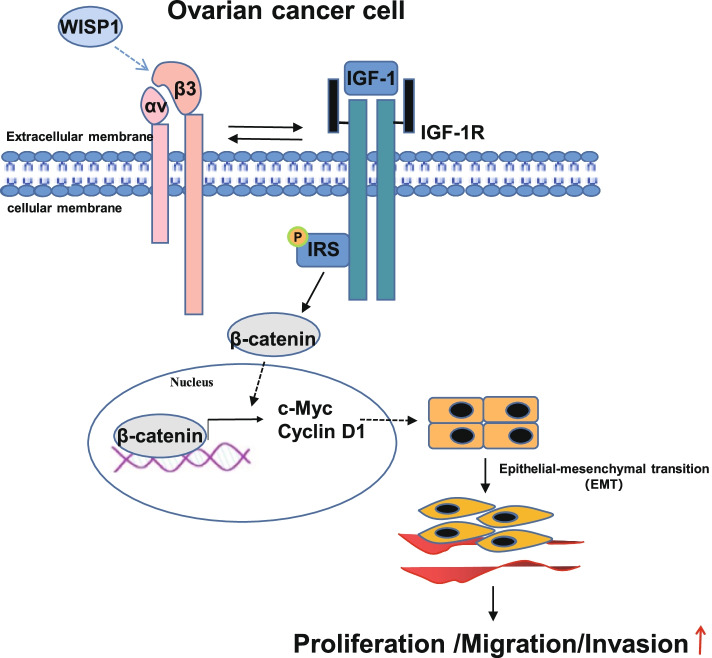
WISP1 activates the Wnt signal axis by promoting the interaction between IGF1 and αvβ3 to enhance EMT, thereby facilitating ovarian cancer

To sum up, our study demonstrated that WISP1 was capable of inducing the interaction between IGF1 and αvβ3 to lead to cell proliferation, migration, invasion, and EMT, thus accelerating ovarian cancer progression. Our findings underline the new therapeutic direction for limiting ovarian cancer (Fig. 6). Due to the limited research, the roles of WISP1, IGF1, αvβ3, and Wnt as well as their interaction in the progression of ovarian cancer should be more clearly investigated. In addition, the WISP1/IGF1/αvβ3 signaling axis is mainly completed in the in vitro cell line. Even if we use the mouse xenograft model and lung metastasis model, it still exists many limitations, including the significantly lower heterogeneity of the in vitro cell line than that of the tumor cells in the tumor tissue, and the difference between the microenvironment in the mouse and the microenvironment in the human body (the immune deficient mice basically have no immune system and other problems). Therefore, it is necessary to further explore the importance of this signaling axis in mouse orthotopic tumor model, and even in clinic.

## Supplementary Information

**Additional file 1: Supplementary Fig. 1.** A Representative protein bands of Fig. 2E. B Representative protein bands of Fig. 2H. C Representative protein bands of Fig. 2I. D Representative protein bands of Fig. 2J. E Representative protein bands of Fig. 3E. F Representative protein bands of Fig. 3H. G Representative protein bands of Fig. 3I. H Representative protein bands of Fig. 3J. I Representative protein bands of Fig. 4G. J Representative protein bands of Fig. 4J. K Representative protein bands of Fig. 4K.

**Additional file 2.**

## Data Availability

The data underlying this article will be shared on reasonable request to the corresponding author.
