# Supplementary material for: WISP1 induces ovarian cancer via the IGF1/αvβ3/Wnt axis
Source: J Ovarian Res. 2022 Aug 13;15:94. doi: 10.1186/s13048-022-01016-x (PMC9375285; doi:10.1186/s13048-022-01016-x)
Supplement: Supplementary file 2 — Additional file 2. [file 13048_2022_1016_MOESM2_ESM.docx]

**Supplementary Table 1** shRNA primer sequences

| shRNA | Sequence |
| --- | --- |
| shNC  shIGF1#1 | SHC007 (Sigma)  CAAGAACTACAGGATGTAGGA |
| shIGF1#2  shIGF1#3 | CACAAATGCATGGGTGTTGTA  CCAATTATTTAAGTGCTGCTT |
| shWISP1#1  shWISP1#2  shWISP1#3 | GCATCCATGAACTTCACACTT  CCACTCGGATCTCCAATGTTA  CTGTGGAGTTTGCATGGACAA |

**Note:** shRNA, short hairpin RNA; NC, negative control; IGF1, insulin growth factor-1; WISP1, WNT1 inducible signaling pathway protein 1.

**Supplementary Table 2** RT-qPCR primer sequences

| Gene | Forward | Reverse |
| --- | --- | --- |
| *GAPDH* | GTCTCCTCTGACTTCAACAGCG | ACCACCCTGTTGCTGTAGCCAA |
| *IGF1* | CTCTTCAGTTCGTGTGTGGAGAC | CAGCCTCCTTAGATCACAGCTC |
| *WISP1* | AAGAGAGCCGCCTCTGCAACTT | TCATGGATGCCTCTGGCTGGTA |
| *CDH1* | GCCTCCTGAAAAGAGAGTGGAAG | TGGCAGTGTCTCTCCAAATCCG |
| *TJP1* | GTCCAGAATCTCGGAAAAGTGCC | CTTTCAGCGCACCATACCAACC |
| *CDH2* | CCTCCAGAGTTTACTGCCATGAC | GTAGGATCTCCGCCACTGATTC |
| *FN1* | ACAACACCGAGGTGACTGAGAC | GGACACAACGATGCTTCCTGAG |
| *CDH11* | GATCGTCACACTGACCTCGACA | CTTTGGCTTCCTGATGCCGATTG |
| *MMP2* | AGCGAGTGGATGCCGCCTTTAA | CATTCCAGGCATCTGCGATGAG |
| *SNAIL* | TGCCCTCAAGATGCACATCCGA | GGGACAGGAGAAGGGCTTCTC |
| *SLUG* | ATCTGCGGCAAGGCGTTTTCCA | GAGCCCTCAGATTTGACCTGTC |

Note: RT-qPCR, reverse transcription quantitative polymerase chain reaction; GAPDH, Glyceraldehyde-3-phosphate dehydrogenase; IGF1, insulin growth factor-1; WISP1, WNT1 inducible signaling pathway protein 1; CDH1, cadherin 1; TJP1, tight junction protein 1; FN1, fibronectin 1; MMP2, Matrix metalloproteinase-2.

**Supplementary Table 3** Name and source of antibody

| Antibody | Company | Article number |
| --- | --- | --- |
| E-cadherin | Abcam | ab40772 (1: 10000) |
| N-cadherin | Abcam | ab18203 (1: 1000) |
| Snail | Thermo Fisher | PA5-115940 (1: 100) |
| Slug | Abcam | ab27568 (1: 500) |
| IGF1 | Abcam | ab9572 (1: 1000) |
| TGF-β | Abcam | ab215715 (1: 1000) |
| P-SMAD2 (S-467) | Abcam | ab53100 (1: 1000) |
| SMAD2 | Abcam | ab33875 (1: 1000) |
| P-PI3K (Y607) | Abcam | ab182651 (1: 500) |
| PI3K | Abcam | ab191606 (1: 1000) |
| P-AKT (T308) | Abcam | ab38449 (1: 500) |
| AKT | Abcam | ab8805 (1: 500) |
| β-catenin | Abcam | ab32572 (1: 5000) |
| C-Myc | Abcam | ab32072 (1: 1000) |
| Cyclin D1 | Abcam | ab40754 (1: 1000) |
| WISP1 | Thermo Fisher | PA5-18832 (1: 1000) |
| P-IGF1R | Abcam | ab39398 (1: 1000) |
| IGF1R | Abcam | ab182408 (1: 1000) |
| P-IRS1 | Abcam | ab4776 (1: 1000) |
| IRS1 | Abcam | ab40777 (1: 1000) |
| ZO-1 | Abcam | ab221546 (1: 1000) |
| GAPDH | Abcam | ab9484 (1: 2000) |
| Goat Anti-Rabbit IgG H&L | Abcam | ab6721 (1: 2000) |
| Goat Anti-Mouse IgG H&L | Abcam | ab205719 (1: 2000) |

**Supplementary Table 4** Demographic and pathological information of patients with ovarian cancer

| Pathological index | Percentage % |
| --- | --- |
| Median age (range) | 55 (34-76) |
| Histopathological diagnosis |  |
| Mucinous ovarian cancer | 29/57 (50.88) |
| Endometrioid ovarian cancer | 10/57 (17.54) |
| Serous adenocarcinoma | 18/57 (31.58) |
| Grade of ovarian cancer |  |
| Well differentiated | 9/57 (19.30) |
| Moderately differentiated | 23/57 (35.09) |
| Poorly differentiated | 50/57 (45.61) |

**Supplementary Table 5** IGF1-related genes

| Genes | Pearson-CC |
| --- | --- |
| MMP2 | 0.54 |
| COL1A1 | 0.54 |
| COL1A2 | 0.51 |
| CDH11 | 0.49 |
| ZEB2 | 0.47 |
| ITGA5 | 0.42 |
| FZD1 | 0.39 |
| MRC2 | 0.38 |
| SNAI2 | 0.38 |
| FN1 | 0.36 |
| TNS1 | 0.35 |
| PDGFRB | 0.35 |
| PLAU | 0.33 |
| SNAI1 | 0.32 |
| TGFBR1 | 0.30 |
| PLAUR | 0.30 |
| ITGB1 | 0.30 |
